# Is Traditional Chinese Medicine “Mainstream” in China? Trends in Traditional Chinese Medicine Health Resources and Their Utilization in Traditional Chinese Medicine Hospitals from 2004 to 2016

**DOI:** 10.1155/2020/9313491

**Published:** 2020-05-31

**Authors:** Xuefeng Shi, Dawei Zhu, Stephen Nicholas, Baolin Hong, Xiaowei Man, Ping He

**Affiliations:** ^1^School of Management, Beijing University of Chinese Medicine, Beijing 100029, China; ^2^National Institute of Chinese Medicine Development and Strategy, University of Chinese Medicine, Beijing 100029, China; ^3^China Center for Health Development Studies, Peking University, Beijing 100191, China; ^4^Australian National Institute of Management and Commerce, Sydney, NSW 2015, Australia; ^5^Research Institute for International Strategies, Guangdong University of Foreign Studies, Guangdong 510420, China; ^6^School of Economics and School of Management, Tianjin Normal University, Tianjin 300074, China; ^7^Newcastle Business School, University of Newcastle, University Drive, Newcastle, NSW, Australia

## Abstract

**Background:**

Traditional, complementary, and alternative medicine (TCAM) has attracted increasing attention in developed countries, but its mainstream status in China, the home of TCAM, is unclear. Over the period of 2004–2016, we analyze the health resources and health resource utilization of traditional medicine in traditional Chinese medicine (TCM) hospitals in China.

**Methods:**

Over 2004–2016, we obtained data from all TCM hospitals in all Chinese provinces to create a hospital-based, longitudinal dataset. TCM health resources and their utilization were measured by two outcome variables: (1) primary outcome variables comprising the proportion of TCM physicians, TCM pharmacists, revenue from TCM drugs, and TCM prescriptions and (2) the secondary outcome variables, as proxies of westernization for TCM hospitals, comprising the number of medical equipment above RMB 10,000 and the proportion of surgery in inpatient visits. We used linear regression models with hospital-fixed effects to analyze time trends for the outcome variables.

**Results:**

The number of public TCM hospitals remained stable from 2004 to 2016, while the number of private TCM hospitals increased from 294 in 2004 to 1560 in 2016. There was a small percentage increase in the proportion of TCM physicians (0.280%), TCM pharmacists (0.298%), and revenue from Chinese medicines (0.331%) and TCM prescriptions (1.613%) per hospital per year. Chinese drugs accounted for less than a half of the total drug prescriptions, and accordingly, just one-third of the drug revenue was from Chinese medicines at TCM hospitals. The proportions of physicians, pharmacists, revenue from Chinese drug sales, and traditional medicine prescriptions never reach the 60% benchmark target for mainstream in TCM hospitals. As proxies for Western medicine practices in TCM hospitals, the number of medical equipment above RMB 10,000 rapidly rose by over 13 percent per hospital per year, but the proportion of inpatient surgeries declined by 0.830 percentage points per hospital per year, reflecting a mixed trend in the use of Western medicine practices.

**Conclusion:**

For the 2004–2016 period, traditional medicine, although making progress towards the mainstream benchmark of 60% TCM services, was still not mainstream at TCM hospitals.

## 1. Background

In developed countries, there has been renewed interest in traditional, complementary, and alternative medicine (TCAM) [1, 2], with the “World Health Organization Traditional Medicine Strategy 2014–2023,” promoting appropriate TCAM policies in support of traditional medicine [3]. Cross-national studies report a substantially increased use of TCAM worldwide [4, 5]. In the United States, National Health Interview Surveys found that the 12-month prevalence of TCAM use by adults and children climbed from 28.9% in 1999 to 38.3% in 2007 [6]. In Australia, the South Australian Health Omnibus Surveys showed a higher utilization of TCAM, with the prevalence of 48.5% in 1993 [7], 52.1% in 2006 [8], and 52.2% in 2004 [9]. As a part of this growing trend, traditional Chinese medicine (TCM), which is one of the oldest alternative healing options around the world [10], has also gained increased application in Western societies [11].

Surprisingly, the picture is less clear in the home of TCAM, China. In the early 1950s, TCM was an ideological and practical component in China's health care system [12], but China's 1970s market reform and opening-up saw the use of TCM as contrary in a society committed to modernization [13]. From 1991 to 2004, the percentage of people consulting TCM doctors in the formal health sectors declined from 25% to 20% in urban China and from 14% to 16% in rural China [14].

But, TCM is central to Chinese health provision, with few studies published in English on the trend of TCM use in mainland China, especially given the national health care reforms since 2009. China is the only country where both Western medicine and TCM are practiced alongside each other in hospitals and primary care facilities [15]. TCM has its own department at the Ministry of Health and at provincial and county bureaus of health, as well as its own medical schools, hospitals, and research institutes [12]. Among these, TCM hospitals were the main providers of TCM services, which combine the use of medicinal herbs, moxibustion, acupuncture, dietary therapy, massage, and therapeutic mind/body practices [16, 17]. In TCM hospitals, TCM and modern medicine are practiced together to improve human health [15].

According to government regulations, TCM hospitals should maintain their mainstream status as Chinese medicine providers in terms of human resource allocation, department structure, and health care delivery [18]. However, modern medicine practices posed dilemmas for TCM, including the lack of TCM practitioners and unwillingness of hospitals to provide TCM services [19]. In contrast, the Chinese government has a long history of supporting the development of TCM. This was confirmed in the 2016 Outline of the Healthy China 2030 Plan [20], which guided the improvement in China's national health by setting out a series of measures to support and develop TCM. These measures included advancing the historical place of TCM in health care, improving the ability to provide TCM services, and promoting innovation in TCM. Given the unique role of TCM in China, whether TCM services were mainstream in health care delivery at TCM hospitals is of great importance to understanding health care provision and policy in China.

Constructing a longitudinal dataset from all TCM hospitals in China from 2004 to 2016, the aim of this study is to analyze the mainstream status of TCM in TCM hospitals and map the 2004–2016 TCM trends in human resources, equipment allocation, service provision, and revenue structure. Our results provide data to assess the development of traditional medicine in China and promote traditional medicine in other low-income countries, where a majority of the population rely on traditional medicine.

## 2. Methods

### 2.1. Data Source

**Figure 1:**
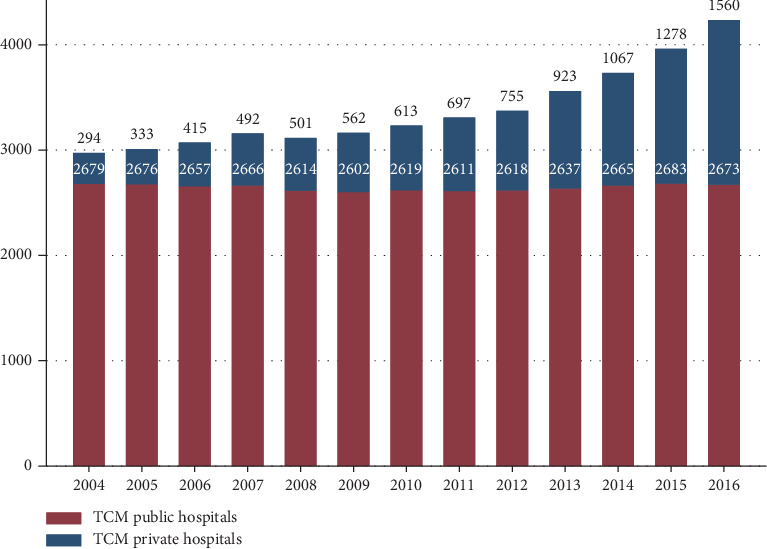
Number of TCM hospitals in China from 2004–2016.

Based on data from over 3000 TCM hospitals during 2004–2016 (43,890 hospital-years), this longitudinal study analyzes the trends in TCM health resources and their utilization in China. To attenuate sampling errors, we obtained data from all TCM hospitals in all mainland Chinese provinces between 2004 and 2016 to establish a hospital-based, longitudinal dataset for analysis. These data, collected annually by the National Health Committee of the People's Republic of China, covered both public and private TCM hospitals and consisted of information on human resources, equipment, medicine prescriptions, revenues, and outpatient and inpatient services. TCM hospitals included primary, secondary, and tertiary hospitals, as well as public and private hospitals. As shown in Figure 1, the number of public TCM hospitals remained stable from 2004 to 2016, but the number of private TCM hospitals grew from 294 in 2004 to 1560 in 2016.

### 2.2. Measurements

The Basic Standards for Medical Institutions [21], which was enacted in 1994 and revised in 2017, stipulated that TCM health professionals shall represent at least 60% of the total health professionals at TCM hospitals. Based on this rubric, we defined “mainstream status” as TCM health resources, and their utilization in TCM hospitals should account for at least 60 percent of all health resources and their utilization. TCM health resources and their utilization were measured by the proportion of TCM physicians in all physicians, the proportion of TCM pharmacists in all pharmacists, the proportion of TCM prescriptions in all prescriptions, and the proportion of revenue from TCM practices in all revenue. The higher (lower) the values of these human resources, prescriptions, and revenue measures, the more (less) successful the TCM mainstream practices in TCM hospitals. To assess the underlying tradeoff between TCM and Western medicine, the quantity of medical equipment above RMB 10,000 and the percentage of inpatients undergoing surgery were used to measure Western medical practices in TCM hospitals. The larger the number of these Western practices, the lower the mainstream status of TCM practices in TCM hospitals.

For the 2004–2016 period, we collected data on TCM physicians and pharmacists, revenue from Chinese medicines, the quantity of equipment above RMB 10,000, and the number of inpatient surgeries. Due to data restrictions, we obtained data on TCM pharmacists from 2008 to 2016 and TCM prescriptions from 2012 to 2016.

Year was defined as a continuous independent variable, which was used to identify the time trend and annual average change in each outcome variable. In addition, we also treated year as a categorical variable to observe the year-by-year trend of each outcome.

### 2.3. Statistical Analysis

Descriptive statistical analysis was performed, and time-trend analysis for outcome variables was conducted using linear regression models with hospital-fixed effects. An advantage of the fixed effects model is that it controls for time-invariant heterogeneity among hospitals, such as underlying aspects of hospital culture.

Initially, year was included in the model as a continuous variable to estimate the average annual changes in the characteristics of traditional medicine at TCM hospitals. The size of the coefficient for each outcome gave us the size of average annual changes on that outcome variable over the 2004–2016 study period.

Since private TCM hospitals developed rapidly and played an increasing role in China's health system [22], we then included interactions between hospital ownership and continuous year to analyze the average annual changes by hospital ownership.

Finally, replacing the continuous year variable with year dummies, we analyzed the year-by-year trend on the features of traditional medicine at TCM hospitals by hospital ownership. These fixed effects models provided estimations of year-by-year changes in traditional medicine practices at private TCM hospitals and whether there was a statistically significant difference in the changes in TCM practices between private and public TCM hospitals.

Standard errors are clustered at the prefecture level. A *p* value of less than 0.05 was considered statistically significant. The software STATA version 15 for Windows (StataCorp, College Station, TX, USA) was used for the statistical analysis.

## 3. Results

### 3.1. Characteristics of TCM Hospitals

Table 1 displays the characteristics of TCM hospitals. Overall, TCM physicians accounted for 45.1% of all physicians, and TCM pharmacists accounted for 51.7% of the total pharmacists per hospital per year in TCM hospitals from 2004 to 2016. The average proportion of revenue from Chinese medicines in all drug revenue was 33.8%, and the mean proportion of TCM prescriptions in all prescriptions was 46.6%. The average number of medical equipment above RMB 10,000 was significantly smaller in private (19.5) than public (114.1) hospitals, and Chinese drug revenue and traditional medicine prescriptions formed a significantly larger share of all revenue and prescriptions in private compared to public hospitals. The average percentage of inpatients undergoing surgery was 21.8%.

### 3.2. Average Changes in Health Resource and Utilization of Traditional Medicine at TCM Hospitals

**Figure 2:**
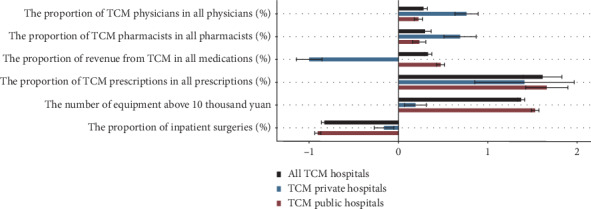
Average changes on the mainstream status of traditional medicine at TCM hospitals in 2004–2016.

Figure 2 presents the average changes in health resources and the utilization of traditional medicine at TCM hospitals by hospital ownership from 2004 to 2016, where the error bars are confidence intervals. Corresponding regression results are presented in Supplementary Tables 1 and 2. Figure 2 displays the increment of percentage points of TCM physicians (0.280), TCM pharmacists (0.298), revenue from Chinese drugs (0.331), and TCM prescriptions (1.613) per hospital per year. The number of medical equipment above RMB 10,000 rapidly rose by over 13 percentage points per hospital per year. The proportion of inpatients undergoing surgery declined by 0.830 percentage points per hospital per year.

Given the overwhelming number of the public versus private TCM hospitals, stratified analyzes by hospital ownership are presented in Figure 2. The percentage point change of the proportion of TCM physicians and TCM pharmacists per year in TCM public hospitals was lower than those in TCM private hospitals. The proportion of revenue from Chinese medicines in all drug revenues in TCM public hospitals increased by 0.473 percentage point as opposed to that in TCM private hospitals, which is decreasing by roughly 1 percentage point. The proportion of TCM prescriptions in all prescriptions in TCM public hospitals (1.41 percent) grew at a rate similar to private hospitals (1.66 percent).

The number of medical equipment above RMB 10,000 at TCM public hospitals increased by more than 15 devices per year, with a growth rate significantly higher than that at TCM private hospitals, which rose by less than 2 devices per year on average. The proportion of inpatient surgeries fell faster at TCM public hospitals (0.903) than private hospitals (0.162).

### 3.3. Year-by-Year Changes on Health Resource and Utilization of Traditional Medicine at TCM Hospitals

**Figure 3:**
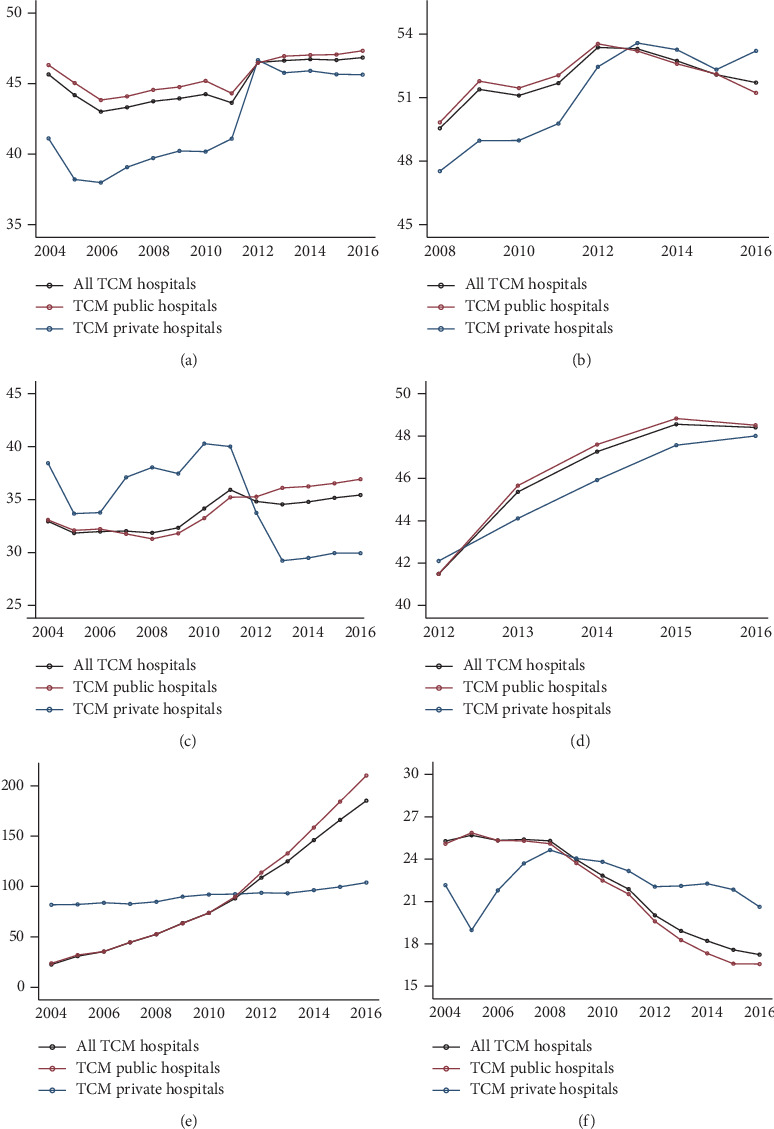
Year-by-year changes on the mainstream status of traditional medicine at TCM hospitals in 2004–2016. (a) The proportion of TCM physicians in all physicians (%). (b) The proportion of TCM pharmacists in all pharmacists (%). (c) The proportion of revenue from Chinese drugs in all drug revenues (%). (d) The proportion of TCM prescriptions in all prescriptions (%). (e) The number of equipment above 10,000 yuan. (f) The proportion of inpatient surgeries.

Figure 3 illustrates the year-by-year trends in health resources and their utilization by hospital ownership. Corresponding regression results are presented in Supplementary Tables 3 and 4. The proportions of TCM physicians among all physicians at TCM public and private hospitals were both below 50% each year in 2004–2016, although TCM private hospitals saw a slight rise in the proportion of TCM physicians before 2012. The proportion of TCM pharmacists in all pharmacists at TCM public and private hospitals both increased from 2008 to 2016, reaching 50% by 2012. The proportion of revenue from Chinese medicines at TCM public hospitals slightly increased over the 2004–2016 period and exceeded 35% of all drug sales by 2011. By contrast, the proportion of revenue of Chinese medicines at TCM private hospitals sharply decreased from about 40% in 2011 to less than 30% in 2013. The proportion of TCM prescriptions at both TCM public and private hospitals gradually rose from 2012, accounting for nearly 50% of the total prescriptions by 2015.

The number of large medical equipment showed a divergent trend between TCM public and private hospitals. While the quantity of large equipment at private hospitals remained stable, the quantity of large equipment at public hospitals surged between 2004 and 2016, surpassing that at private hospitals in 2012. The proportion of inpatient surgeries declined substantially from roughly 25% in 2004 to just above 15% in 2016 at TCM public hospitals, whereas inpatient surgery was below 24% before 2009 and roughly 20% thereafter in TCM private hospitals, as shown in Figure 3.

## 4. Discussion

Our primary aim was to document the trends in traditional medicine health resources and their utilization at TCM hospitals in China by using hospital-based longitudinal data. The Basic Standards for Medical Institutions stipulated that TCM health professionals should represent at least 60% of the total health professionals at TCM hospitals. However, we found that the proportions of TCM physicians and pharmacists, although gradually increasing, never hit the 60% mainstream target during the 2004–2016 period. Chinese medicines, which can only be prescribed by TCM physicians, accounted for, on average, less than a half of the total drug prescriptions in TCM hospitals. Just one-third of the drug revenue was from Chinese medicines at TCM hospitals. In addition, the quantity of large medical equipment, as proxy of westernization for TCM hospitals, grew rapidly during our study period, while the proportion of inpatient surgeries gradually decreased.

In the 2004–2016 period, attaining the mainstream status of traditional medicine at TCM hospitals was not successfully achieved. Since the early 1950s, the Chinese government has promoted the development of TCM practically and ideologically [14]. In the 1980s, the Ministry of Health encouraged the establishment of TCM hospitals, creating a complete network of TCM hospitals, with each province, prefecture city, and county having their own TCM hospitals. How to balance Chinese medicine and Western medicine at TCM hospitals has been a long and serious concern of health policy planners. Early in the 1990s, scholars started criticizing the westernization of TCM hospitals, where Western health care services have been increasingly provided due to their higher profit relative to TCM treatments [23]. Despite the Basic Standards for Medical Institutions setting a 60% TCM physician and pharmacist requirement, the required standard was not met during our study period. Three reasons may account for this phenomenon. First, routine evaluation on the features of traditional medicine for TCM hospitals by the State Administration of Traditional Chinese Medicine since 2005 has only made limited progress in incrementally raising the proportion of TCM physicians and pharmacists. Second, a majority of health professionals, not only Western physicians but also TCM physicians and health care mangers, viewed TCM only as a complement to Western medicine [24]. Third, recruiting qualified TCM physicians was challenging because the expectations of TCM students were lower than Western medicine students in terms of lower career development and salary [25, 26]. Consequently, TCM hospitals experienced insufficient TCM trainees.

The proportion of prescription and revenue from Chinese medicines gradually rose, but did not attain the 60% mainstream target at TCM hospitals. As the core component of the 2009 public hospital reform, the zero-markup drug policy (ZMDP) imposed a zero profit margin on Western drug sales at county hospitals in 2012, county hospitals in 2015, and tertiary hospitals in 2017. To control excess drug prescriptions, ZMDP stopped the usual 15% markup on Western medicines, but exempted Chinese-patented drugs. Our data reflect the TCM hospitals' financial motivation for prescribing Chinese medicine and the disincentive from prescribing Western drugs, with the proportion of prescriptions and revenue from Chinese drugs increasing. In addition, the growth of TCM physicians, although not as fast as expected, also meant more prescriptions of Chinese medicines. However, the volume of prescriptions and revenue from Chinese medicines remained in the minority at TCM hospitals, which is consistent with the previous research [27].

As measures of Western medical practices, the number of inpatient surgeries declined at TCM hospitals, but the quantity of large medical equipment increased over time. Early in the 1990s, the revenue of TCM hospitals heavily relied on surgeries due to their higher profit margins [28]. Recent studies confirmed that TCM hospitals delivered an increasing number of surgeries with more expensive and advanced medical equipment from 2000 to 2008 [29]. In our study, we found a significant downward trend in inpatient surgeries at TCM hospitals. In part, this reflects the fruits of regular monitoring and evaluation to maintain the mainstream status of traditional medicine at TCM hospitals. Second, the 2009 health reforms aimed at reducing overservicing, which discouraged unnecessary surgeries, especially at TCM hospitals. However, we observed an upward tendency in the quantity of large medical equipment at TCM hospitals. This is probably due to the policy of government health investment in fixed assets, including medical equipment, rather than human resources [30]. Since 2009, the Chinese government rapidly increased the public investment in public hospitals, of which a large proportion was used to purchase medical equipment.

Among multiple variables impacting traditional health resources and their utilization, there were some different private-public TCM hospital trends, but mainly, the trends moved in common. Since the policy of attaching the equal importance to Western medicine and Chinese traditional medicine was put forward at the 17th CPC National Congress in 2007, which was further emphasized during China's health system reform in 2009, the Chinese government has implemented policies to promote the development of TCM and to attract private funding to establish TCM private hospitals [31]. The number of private TCM hospitals increased during 2004–2016, but the scale of private TCM hospitals was only about 20% of public TCM hospitals, with public hospitals remaining the overwhelming majority in the TCM medical providers. Both public and private TCM hospitals saw an increased proportion of TCM physicians, pharmacists, and prescriptions, as well as a decrease in the proportion of inpatient surgeries. However, differentiated investment and stewardship policies led to differences in the resources and services delivery between public and private TCM hospitals. For example, the majority of government health investment was distributed to public hospitals, resulting in a surge in the volume of medical equipment at public TCM hospitals rather than private TCM hospitals. In addition, the ZMDP, eradicating add-on profits of prescribing Western medicines, was implemented in public hospitals but not in private hospitals. This meant that public TCM hospitals were only allowed to generate markup profits from traditional medicines, and private TCM hospitals were allowed to make profits by prescribing both Western and Chinese medicines. This likely explains why the proportion of revenue from Chinese medicines showed an upward trend at public TCM hospitals, but a downward trend at private TCM hospitals.

This study is subject to several limitations. The present study only focused on supply-side factors to investigate to what extent TCM hospitals kept the mainstream status of traditional medicine. We did not investigate demand-side factors due to data limitations on individual patients. Future research should document the trends in the use of TCM from the demand side, including collecting data on characteristics of physicians between hospitals with different levels of mainstream status. We were aware of sampling bias, which was addressed by using up-to-date data from all TCM hospitals in mainland China rather than sampling facilities and by covering both public and private hospitals. In addition, we obtained multiple variables to quantify tradition medicine health resources and their utilization in TCM hospitals over a relatively long period.

## 5. Conclusion

For the 2004–2016 period, traditional medicine, although making progress towards the mainstream benchmark of 60% TCM services, was still not mainstream at TCM hospitals. We suggest that the government health policy should be further reviewed to assess its impact on traditional medicine provision at TCM hospitals. Importantly, we recommend demand-side studies to explore the motivation for patients seeking TCM services.

## Figures and Tables

**Table 1:** Characteristics of TCM hospitals in 2004–2016.

		Mean (SD)		
		Public hospitals	Private hospitals	Total
The number of TCM physicians		32.9 (50.8)	7.4 (15.7)	27.4 (46.8)
The proportion of TCM physicians in all physicians (%)		45.7 (22.6)	43.2 (29.8)	45.1 (24.3)
The number of TCM pharmacists		10.0 (14.4)	2.1 (4.3)	8.1 (13.1)
The proportion of TCM pharmacists in all pharmacists (%)		52.4 (25.1)	49.5 (33.2)	51.7 (27.2)
The revenue from Chinese drugs		8.2 (24.4)	1.8 (6.0)	6.8 (22.0)
The proportion of revenue from Chinese drugs in all drug revenues (%)		32.1 (18.4)	40.7 (25.8)	33.8 (20.4)
The number of TCM prescriptions		82.8 (326.4)	7.1 (28.3)	60.4 (276.4)
The proportion of TCM prescriptions in all prescriptions (%)		45.6 (24.4)	50.0 (34.5)	46.6 (27.1)
The number of equipment above 10,000 yuan		114.1 (271.0)	19.5 (55.1)	93.7 (244.5)
The number of inpatient surgeries		1220.9 (2246.4)	311.5 (910.5)	1023.4 (2066.6)
The proportion of inpatient surgeries (%)		22.3 (18.0)	19.6 (29.6)	21.8 (20.7)
Observations (hospital-year)		34400	9490	43890

## Data Availability

The data that support the findings of this study are available from the National Health Committee of the People's Republic of China, but restrictions apply to the availability of these data, which were used under license for the current study, and so are not publicly available. Data are, however, available from the authors upon reasonable request and with permission of the National Health Committee of the People's Republic of China.

## Abbreviations

TCM: Traditional Chinese medicine
TCAM: Traditional, complementary, and alternative medicine
ZMDP: Zero-markup drug policy.

## Supplementary Materials

Supplementary Materials Supplementary Table 1: estimated average changes on the mainstream status of traditional medicine at TCM hospitals in 2004–2016. Supplementary Table 2: estimated average changes by ownership on the mainstream status of traditional medicine at TCM hospitals in 2004–2016. Supplementary Table 3: estimated year-by-year changes on the mainstream status of traditional medicine at TCM hospitals in 2004–2016. Supplementary Table 4: estimated year-by-year changes by ownership on the mainstream status of traditional medicine at TCM hospitals in 2004–2016.
