# Supplementary material for: Is Traditional Chinese Medicine “Mainstream” in China? Trends in Traditional Chinese Medicine Health Resources and Their Utilization in Traditional Chinese Medicine Hospitals from 2004 to 2016
Source: Evid Based Complement Alternat Med. 2020 May 31;2020:9313491. doi: 10.1155/2020/9313491 (PMC7281804; doi:10.1155/2020/9313491)
Supplement: Supplementary Materials — Supplementary Table 1: estimated average changes on the mainstream status of traditional medicine at TCM hospitals in 2004–2016. Supplementary Table 2: estimated average changes by ownership on the mainstream status of traditional medicine at TCM hospitals in 2004–2016. Supplementary Table 3: estimated year-by-year changes on the mainstream status of traditional medicine at TCM hospitals in 2004–2016. Supplementary Table 4: estimated year-by-year changes by ownership on the mainstream status of traditional medicine at TCM hospitals in 2004–2016. [file 9313491.f1.docx]

**Supplementary Table 1 Estimated average changes on the mainstream status of traditional medicine at TCM hospitals in 2004-2016**

|  | The share of TCM physicians in all physicians (%) | The share of TCM pharmacist in all pharmacist (%) | The share of revenue from TCM in all medications (%) | The share of TCM prescription in all prescription (%) | The number of equipment above 10 thousand yuan | The share of surgery in inpatient visitors (%) |
| --- | --- | --- | --- | --- | --- | --- |
| Year | 0.280*** | 0.298*** | 0.331*** | 1.613*** | 13.738*** | -0.830*** |
|  | (0.022) | (0.035) | (0.022) | (0.111) | (0.209) | (0.017) |
| Observations | 43,319 | 32,678 | 42,307 | 12,881 | 43,877 | 39,802 |
| R-squared | 0.004 | 0.003 | 0.006 | 0.023 | 0.102 | 0.063 |

**NOTES** Model was adjusted for hospital fixed effects. Standard errors which are clustered at the prefecture level in parentheses. *** p<0.001, ** p<0.01, * p<0.05.

**Supplementary Table 2 Estimated average changes by ownership on the mainstream status of traditional medicine at TCM hospitals in 2004-2016**

|  | The share of TCM physicians in all physicians (%) | The share of TCM pharmacist in all pharmacist (%) | The share of revenue from TCM in all medications (%) | The share of TCM prescription in all prescription (%) | The number of equipment above 10 thousand yuan | The share of surgery in inpatient visitors (%) |
| --- | --- | --- | --- | --- | --- | --- |
| Non-Public TCM hospital*Year | 0.762*** | 0.690*** | -0.999*** | 1.411*** | 1.914** | -0.162** |
|  | (0.066) | (0.092) | (0.073) | (0.284) | (0.620) | (0.056) |
| Public TCM hospital*Year | 0.224*** | 0.233*** | 0.473*** | 1.661*** | 15.305*** | -0.903*** |
|  | (0.024) | (0.038) | (0.023) | (0.121) | (0.221) | (0.018) |
| Observations | 43,271 | 32,634 | 42,270 | 12,852 | 43,823 | 39,770 |
| R-squared | 0.006 | 0.003 | 0.016 | 0.023 | 0.112 | 0.068 |

**NOTES** Model was adjusted for hospital fixed effects. Standard errors which are clustered at the prefecture level in parentheses. *** p<0.001, ** p<0.01, * p<0.05.

**Supplementary Table 3 Estimated year-by-year changes on the mainstream status of traditional medicine at TCM hospitals in 2004-2016**

|  | The share of TCM physicians in all physicians (%) | The share of TCM pharmacist in all pharmacist (%) | The share of revenue from TCM in all medications (%) | The share of TCM prescription in all prescription (%) | The number of equipment above 10 thousand yuan | The share of surgery in inpatient visitors (%) |
| --- | --- | --- | --- | --- | --- | --- |
| 2005 | -1.468(0.397) *** | - | -1.106(0.413) ** | - | 8.298(3.743) * | 0.412(0.314) |
| 2006 | -2.637(0.397) *** | - | -0.966(0.413) * | - | 12.946(3.740) *** | 0.047(0.314) |
| 2007 | -2.326(0.397) *** | - | -0.925(0.405) * | - | 21.984(3.741) *** | 0.117(0.310) |
| 2008 | -1.899(0.400) *** | -0.148(0.425) | -1.086(0.408) ** | - | 30.043(3.763) *** | 0.015(0.312) |
| 2009 | -1.699(0.399) *** | 1.689(0.427) *** | -0.606(0.406) | - | 41.185(3.756) *** | -1.312(0.311) *** |
| 2010 | -1.399(0.398) *** | 1.402(0.427) ** | 1.211(0.404) ** | - | 51.216(3.745) *** | -2.451(0.310) *** |
| 2011 | -2.007(0.397) *** | 1.985(0.426) *** | 2.982(0.403) *** | - | 65.622(3.731) *** | -3.407(0.309) *** |
| 2012 | 0.851(0.396) * | 3.674(0.425) *** | 1.879(0.401) *** | - | 86.239(3.722) *** | -5.255(0.309) *** |
| 2013 | 0.980(0.393) * | 3.604(0.423) *** | 1.602(0.399) *** | 3.873(0.493) *** | 102.496(3.697) *** | -6.367(0.307) *** |
| 2014 | 1.078(0.392) ** | 3.037(0.422) *** | 1.839(0.398) *** | 5.775(0.489) *** | 123.506(3.684) *** | -7.071(0.306) *** |
| 2015 | 1.021(0.392) ** | 2.384(0.422) *** | 2.219(0.398) *** | 7.068(0.488) *** | 143.607(3.681) *** | -7.705(0.306) *** |
| 2016 | 1.194(0.394) ** | 2.010(0.426) *** | 2.487(0.401) *** | 6.919(0.493) *** | 162.688(3.707) *** | -8.044(0.308) *** |
| Constant | 45.647(0.291) *** | 49.705(0.311) *** | 32.948(0.301) *** | 41.489(0.374) *** | 22.510(2.737) *** | 25.282(0.229) *** |
| Observations | 43,319 | 32,678 | 42,307 | 12,881 | 43,877 | 39,802 |
| R-squared | 0.009 | 0.006 | 0.009 | 0.028 | 0.107 | 0.069 |

**NOTES** Model was adjusted for hospital fixed effects. Standard errors which are clustered at the prefecture level in parentheses. *** p<0.001, ** p<0.01, * p<0.05.

**Supplementary Table 4 Estimated year-by-year changes by ownership on the mainstream status of traditional medicine at TCM hospitals in 2004-2016**

|  | The share of TCM physicians in all physicians (%) | The share of TCM pharmacist in all pharmacist (%) | The share of revenue from TCM in all medications (%) | The share of TCM prescription in all prescription (%) | The number of equipment above 10 thousand yuan | The share of surgery in inpatient visitors (%) |
| --- | --- | --- | --- | --- | --- | --- |
| 2005 | -2.906(1.293) * | - | -4.763(2.136) * | - | 0.324(12.031) | -3.182(1.166) ** |
| 2006 | -3.128(1.275) * | - | -4.664(2.094) * | - | 1.990(11.818) | -0.372(1.143) |
| 2007 | -2.038(1.259) | - | -1.331(1.922) | - | 0.820(11.732) | 1.544(1.098) |
| 2008 | -1.389(1.267) | -0.007(1.134) | -0.398(1.914) | - | 2.986(11.797) | 2.489(1.105) * |
| 2009 | -0.888(1.255) | 1.436(1.137) | -0.982(1.902) | - | 7.982(11.682) | 1.893(1.094) |
| 2010 | -0.933(1.248) | 1.445(1.132) | 1.844(1.898) | - | 10.188(11.610) | 1.655(1.091) |
| 2011 | -0.021(1.235) | 2.243(1.117) * | 1.570(1.887) | - | 10.560(11.487) | 1.003(1.082) |
| 2012 | 5.548(1.230) *** | 4.923(1.110) *** | -4.697(1.882) * | - | 11.872(11.443) | -0.107(1.082) |
| 2013 | 4.651(1.218) *** | 6.049(1.095) *** | -9.210(1.875) *** | 2.009(1.355) | 11.399(11.338) | -0.055(1.072) |
| 2014 | 4.790(1.213) *** | 5.731(1.090) *** | -8.950(1.871) *** | 3.825(1.335) ** | 14.498(11.291) | 0.107(1.067) |
| 2015 | 4.543(1.209) *** | 4.793(1.085) *** | -8.489(1.869) *** | 5.469(1.336) *** | 17.759(11.252) | -0.316(1.064) |
| 2016 | 4.517(1.213) *** | 5.675(1.094) *** | -8.502(1.872) *** | 5.906(1.350) *** | 22.048(11.291) | -1.538(1.067) |
| Public | 5.201(1.393) *** | 2.437(1.473) | -5.358(1.967) ** | -0.611(2.783) | -58.128(12.996) *** | 2.930(1.174) * |
| 2005*Public | 1.633(1.359) | - | 3.778(2.176) | - | 7.926(12.654) | 3.962(1.211) ** |
| 2006*Public | 0.649(1.342) | - | 3.806(2.136) | - | 9.745(12.455) | 0.606(1.189) |
| 2007*Public | -0.183(1.328) | - | 0.012(1.967) | - | 20.130(12.381) | -1.335(1.145) |
| 2008*Public | -0.373(1.336) | -0.132(1.224) | -1.393(1.961) | - | 25.914(12.454) * | -2.483(1.153) * |
| 2009*Public | -0.669(1.326) | 0.373(1.227) | -0.287(1.949) | - | 31.672(12.347) * | -3.255(1.143) ** |
| 2010*Public | -0.192(1.319) | 0.040(1.223) | -1.679(1.945) | - | 39.835(12.278) ** | -4.255(1.140) *** |
| 2011*Public | -1.983(1.307) | -0.155(1.210) | 0.565(1.934) | - | 55.530(12.164) *** | -4.568(1.131) *** |
| 2012*Public | -5.399(1.302) *** | -1.349(1.204) | 6.888(1.929) *** | - | 78.234(12.124) *** | -5.382(1.131) *** |
| 2013*Public | -4.023(1.291) ** | -2.816(1.190) * | 12.233(1.923) *** | 2.161(1.458) | 97.667(12.026) *** | -6.765(1.122) *** |
| 2014*Public | -4.084(1.287) ** | -3.102(1.185) ** | 12.113(1.919) *** | 2.287(1.438) | 120.257(11.980) *** | -7.871(1.116) *** |
| 2015*Public | -3.800(1.283) ** | -2.650(1.182) * | 11.948(1.917) *** | 1.871(1.439) | 142.756(11.951) *** | -8.179(1.115) *** |
| 2016*Public | -3.506(1.289) ** | -4.420(1.192) *** | 12.345(1.921) *** | 1.118(1.454) | 164.368(11.999) *** | -6.979(1.118) *** |
| Constant | 41.114(1.257) *** | 47.537(1.251) *** | 38.440(1.882) *** | 42.101(2.258) *** | 81.871(11.712) *** | 22.159(1.088) *** |
| Observations | 43,271 | 32,634 | 42,270 | 12,852 | 43,823 | 39,770 |
| R-squared | 0.011 | 0.007 | 0.026 | 0.028 | 0.120 | 0.075 |

**NOTES** Model was adjusted for hospital fixed effects. Standard errors which are clustered at the prefecture level in parentheses. *** p<0.001, ** p<0.01, * p<0.05.
